# Calixarene-based mol­ecular capsule from olefin metathesis

**DOI:** 10.1107/S1600536813014438

**Published:** 2013-06-08

**Authors:** Shimelis T. Hailu, Ray J. Butcher, Paul F. Hudrlik, Anne M. Hudrlik

**Affiliations:** a Department of Chemistry, Howard University, 525 College Street NW, Washington, DC 20059, USA

## Abstract

The reaction of tetra­kis­(all­yloxy)calix[4]arene with the first-generation Grubbs catalyst, followed by catalytic hydrogenation, gave the novel bis-calixarene 15,20,46,51,64,69,74,79-octa­oxatridecacyclo[32.28.8.8^3,28^.1^13,53^.1^22,44^.0^9,14^.0^21,26^.0^38,70^.0^40,45^.0^52,57^.0^59,63^.0^7,80^.0^32,73^]octa­conta-1(63),3,5,7(80),9(14),10,12,21,23,25,28(73),29,31,34,36,38(70),40,42,44,52,54,56,59,61-tetra­cosa­ene benzene monosolvate, C_72_H_72_O_8_·C_6_H_6_. The structure consists of two calix[4]arene units connected by four-carbon chains at each of the four O atoms on their narrow rims, to form a cage. Each of the calix[4]arene units has a flattened cone conformation in which two of the opposite aryl groups are closer together and nearly parallel [dihedral angle between planes = 7.35 (16)°], and the other two aryl groups are splayed outward [dihedral angle between planes = 72.20 (8)°]. While the cavity contains no solvent or other guest mol­ecule, there is benzene solvent mol­ecule in the lattice. Two of the alkyl linking arms were disordered over two conformations with occupancies of 0.582 (3)/0.418 (3) and 0.33 (4)/0.467 (4). They were constrained to have similar metrical and thermal parameters.

## Related literature

For literature related to the use of calixarenes as easily isolable reaction products, see: Asfari *et al.* (2001 ▶); Gutsche (2008 ▶). For literature related to the preparation of bridged calixarenes, see: Yang & Swager (2007 ▶); Hailu *et al.* (2012 ▶). For literature related to the conformation of calixarenes, see: Arduini *et al.* (1995 ▶, 1996 ▶); Drew *et al.* (1997 ▶). For literature related to starting material and catalyst used, see: Ho *et al.* (1996 ▶); Vougioukalakis & Grubbs (2010 ▶).
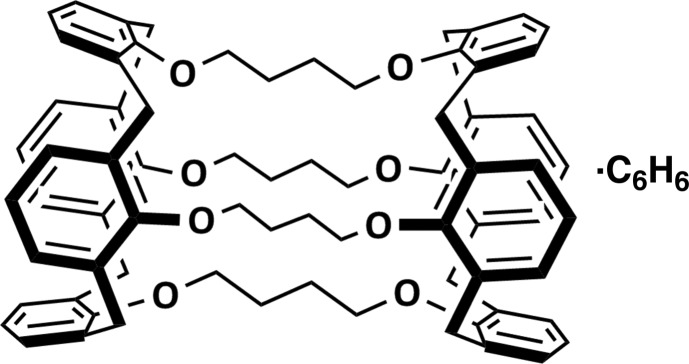


## Experimental

### 

#### Crystal data

C_72_H_72_O_8_·C_6_H_6_
*M* *_r_* = 1143.40
Monoclinic, 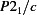

*a* = 14.8804 (10) Å
*b* = 17.3004 (11) Å
*c* = 12.1888 (8) Å
β = 103.929 (7)°
*V* = 3045.6 (3) Å^3^
*Z* = 2
Cu *K*α radiation
μ = 0.62 mm^−1^
*T* = 123 K
0.87 × 0.35 × 0.03 mm

#### Data collection

Agilent Xcalibur (Ruby, Gemini) diffractometer
Absorption correction: analytical (*CrysAlis PRO*; Agilent, 2012 ▶; Clark & Reid, 1995 ▶) *T*_min_ = 0.795, *T*_max_ = 0.982
9972 measured reflections
9972 independent reflections
5548 reflections with *I* > 2σ(*I*)
*R*_int_ = 0.000

#### Refinement

*R*[*F* ^2^ > 2σ(*F* ^2^)] = 0.078
*wR*(*F* ^2^) = 0.249
*S* = 1.00
9972 reflections
415 parameters
10 restraints
H-atom parameters constrained
Δρ_max_ = 1.03 e Å^−3^
Δρ_min_ = −0.24 e Å^−3^

### 

Data collection: *CrysAlis PRO* (Agilent, 2012 ▶); cell refinement: *CrysAlis PRO*; data reduction: *CrysAlis RED* (Agilent, 2012 ▶); program(s) used to solve structure: *SHELXS97* (Sheldrick, 2008 ▶); program(s) used to refine structure: *SHELXL97* (Sheldrick, 2008 ▶); molecular graphics: *SHELXTL* (Sheldrick, 2008 ▶); software used to prepare material for publication: *SHELXTL*.

## Supplementary Material

Crystal structure: contains datablock(s) I, global. DOI: 10.1107/S1600536813014438/hg5315sup1.cif

Structure factors: contains datablock(s) I. DOI: 10.1107/S1600536813014438/hg5315Isup2.hkl

Supplementary material file. DOI: 10.1107/S1600536813014438/hg5315Isup3.cml

Additional supplementary materials:  crystallographic information; 3D view; checkCIF report

## Figures and Tables

**Fig. 1.**
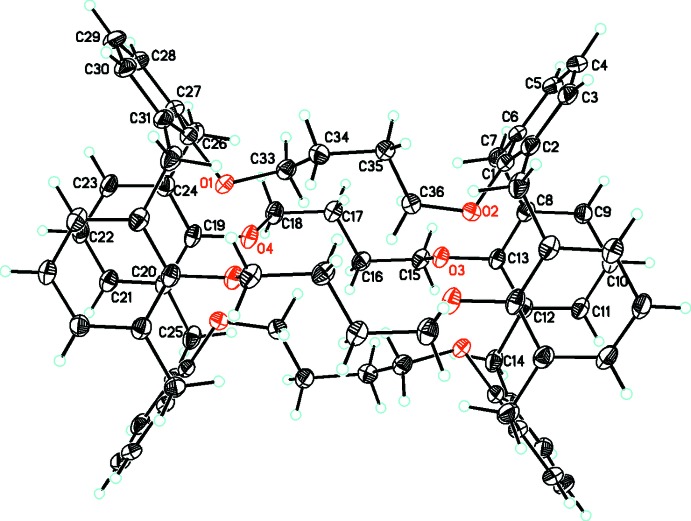
Diagram of C72H72O8 illustrating the atom numbering scheme used. Thermal ellipsoids are at the 30% probability level.

**Fig. 2.**
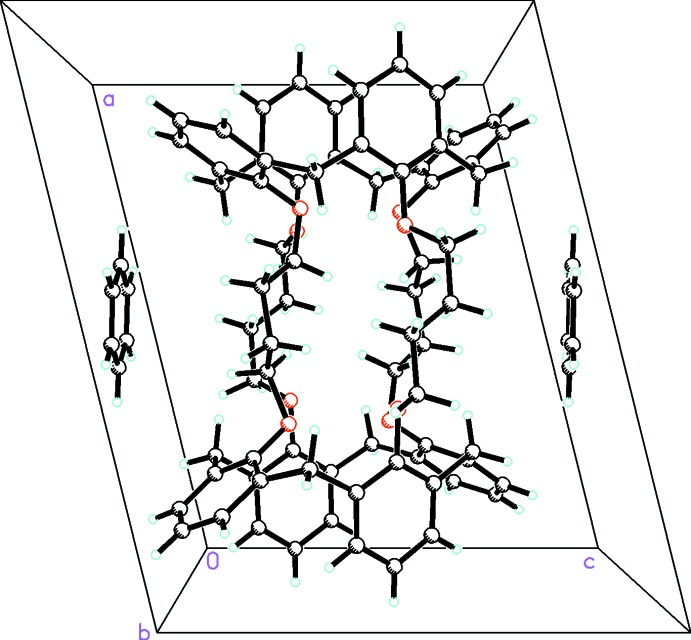
The molecular packing for C72H72O8 viewed down the b axis showing that the benzene solvate is not encapsulated by the calixarene.

## supplementary crystallographic information

### Comment

Calixarenes are macrocyclic molecules made up of phenol and methylene units. The ease of their preparation and chemical modification coupled with the easily isolable reaction products makes them ideal starting materials for the construction of host molecules with different properties (Asfari *et al*., 2001, Gutsche, 2008). Unsubstituted calixarenes have flexible conformations at higher temperatures, and controlling the conformations of calixarenes is an important subject to pursue for better knowledge of the complexing ability of these molecules for various guest ions and molecules. An olefin metathesis reaction (Vougioukalakis & Grubbs, 2010) has been used to prepare bridged calixarenes (Yang & Swager, 2007). In our first attempt to prepare a bridged calixarene by olefin metathesis, the reaction of tetrakis(allyloxy)calix[4]arene with the first generation Grubbs catalyst gave a novel dimeric calixarene with a complex chiral structure (Hailu *et al.*, 2012). In our further investigation of this approach, catalytic hydrogenation of the initial metathesis product gave a small amount of a novel bis-calixarene. In contrast to the chiral structure prepared earlier, the present compound has a very symmetric structure. The two calix[4]arene units which are joined by covalent bonds of (CH_2_)_4_ groups have flattened cone conformations. The degree of flattening of a cone calix[4]arene has been characterized (Arduini *et al.*, 1995; Arduini *et al.*, 1996; Drew *et al.*, 1997) using the dihedral angles between the plane of the four methylene linkers with the phenolic rings. These angles are 83.02 (8)° and 89.67 (8)° for rings B and D, respectively, which are almost parallel to each other, and 145.47 (8)° and 142.32 (7)° for rings A and C, respectively, which are inclined outwards.

Figure 2 shows the molecular packing for the *bis-*calix[4]arene, C_72_H_72_O_8_. The recrystallization solvent, benzene, used in this experiment is shown in the lattice but outside the calixarene cavity.

### Experimental

A 20-mg (0.024 mmol) sample of first-generation Grubbs catalyst measured under nitrogen atmosphere in a glove bag was placed in a 100-ml three-necked flask on a nitrogen line. To this 92 mg (0.157 mmol) of tetrakis(allyloxy)calix[4]arene (Ho *et al.*, 1996) and 40 ml of dichloromethane (distilled from CaH_2_) was added. The mixture was then stirred under reflux in a N_2_ atmosphere at 45 °C (oil bath temperature) for 2 h. Solvent was removed by rotary evaporator to give 92 mg of black solid residue. The residue was then dissolved in 3 ml of dichloromethane and chromatographed (34 g of silica gel, 2.5 *x* 22.5 cm, gradient elution with hexane/dichloromethane). A black residue (presumably catalyst) remained on top of the column, and the fractions were all combined. After removal of solvent by rotary evaporator, a white powder was obtained.

The product was suspended in 7 ml of ethyl acetate and placed on a nitrogen line. Then 20 mg of Pd on powdered charcoal (10%) and 33 ml of ethyl acetate were added while flushing nitrogen through the flask. The flask was then fitted with a stopcock adapter attached to a hydrogen-filled balloon. The connection to the N_2_ line was closed, the stopcock to the hydrogen-filled balloon was opened, and a small amount of hydrogen was allowed to sweep through the flask for few s by slightly opening the glass stopper on one of the necks. The reaction mixture was stirred at room temperature for 6 h. The mixture was then filtered on Celite and solvent removed by rotary evaporator to give 68 mg of white solid product. The product was then chromatographed (8.56 g of silica gel, 1.2 *x* 18 cm, gradient elution with hexane/dichloromethane). Attempted recrystallization of an 8-mg fraction (which showed only one spot on TLC) using CH_2_Cl_2_ and MeOH, and finally benzene gave white crystals suitable for X-ray diffraction analysis.

### Refinement

H atoms were placed in geometrically idealized positions and constrained to ride on their parent atoms with a C—H distance of 0.95 and 0.99 Å *U*_iso_(H) = 1.2*U*_eq_(C).

### Crystal data

**Table:**

C_72_H_72_O_8_·C_6_H_6_	*F*(000) = 1220
*M**_r_* = 1143.40	*D*_x_ = 1.247 Mg m^−^^3^
Monoclinic, *P*2_1_/*c*	Cu *K*α radiation, λ = 1.54178 Å
Hall symbol: -P 2ybc	Cell parameters from 1980 reflections
*a* = 14.8804 (10) Å	θ = 3.1–75.5°
*b* = 17.3004 (11) Å	µ = 0.62 mm^−^^1^
*c* = 12.1888 (8) Å	*T* = 123 K
β = 103.929 (7)°	Plate, colorless
*V* = 3045.6 (3) Å^3^	0.87 × 0.35 × 0.03 mm
*Z* = 2	

### Data collection

**Table:**

Agilent Xcalibur (Ruby, Gemini) diffractometer	9972 independent reflections
Radiation source: Enhance (Cu) X-ray Source	5548 reflections with *I* > 2σ(*I*)
Graphite monochromator	*R*_int_ = 0.000
Detector resolution: 10.5081 pixels mm^-1^	θ_max_ = 75.9°, θ_min_ = 3.1°
ω scans	*h* = −18→18
Absorption correction: analytical (*CrysAlis PRO*; Agilent, 2012; Clark & Reid, 1995)	*k* = −21→21
*T*_min_ = 0.795, *T*_max_ = 0.982	*l* = −15→14
9972 measured reflections	

### Refinement

**Table:**

Refinement on *F*^2^	Primary atom site location: structure-invariant direct methods
Least-squares matrix: full	Secondary atom site location: difference Fourier map
*R*[*F*^2^ > 2σ(*F*^2^)] = 0.078	Hydrogen site location: inferred from neighbouring sites
*wR*(*F*^2^) = 0.249	H-atom parameters constrained
*S* = 1.00	*w* = 1/[σ^2^(*F*_o_^2^) + (0.1443*P*)^2^] where *P* = (*F*_o_^2^ + 2*F*_c_^2^)/3
9972 reflections	(Δ/σ)_max_ < 0.001
415 parameters	Δρ_max_ = 1.03 e Å^−^^3^
10 restraints	Δρ_min_ = −0.24 e Å^−^^3^

### Special details

**Table:**

Experimental. Absorption correction: CrysAlis PRO (Agilent, 2012, Clark & Reid, 1995) Analytical numeric absorption correction using a multifaceted crystal model based on expressions derived by R.C. Clark & J.S. Reid.
Geometry. All e.s.d.'s (except the e.s.d. in the dihedral angle between two l.s. planes) are estimated using the full covariance matrix. The cell e.s.d.'s are taken into account individually in the estimation of e.s.d.'s in distances, angles and torsion angles; correlations between e.s.d.'s in cell parameters are only used when they are defined by crystal symmetry. An approximate (isotropic) treatment of cell e.s.d.'s is used for estimating e.s.d.'s involving l.s. planes.
Refinement. Refinement of *F*^2^ against ALL reflections. The weighted *R*-factor *wR* and goodness of fit *S* are based on *F*^2^, conventional *R*-factors *R* are based on *F*, with *F* set to zero for negative *F*^2^. The threshold expression of *F*^2^ > σ(*F*^2^) is used only for calculating *R*-factors(gt) *etc*. and is not relevant to the choice of reflections for refinement. *R*-factors based on *F*^2^ are statistically about twice as large as those based on *F*, and *R*- factors based on ALL data will be even larger.

### Fractional atomic coordinates and isotropic or equivalent isotropic displacement parameters (Å^2^)

**Table:**

	*x*	*y*	*z*	*U*_iso_*/*U*_eq_	Occ. (<1)
O1	0.30199 (13)	0.55520 (14)	0.31841 (19)	0.0602 (6)	
O2	0.69820 (13)	0.54928 (11)	0.45949 (17)	0.0472 (5)	
O3	0.66583 (12)	0.35803 (11)	0.43693 (17)	0.0492 (5)	
O4	0.33639 (13)	0.36946 (12)	0.32522 (19)	0.0568 (6)	
C1	0.74710 (18)	0.58325 (17)	0.3899 (3)	0.0485 (7)	
C2	0.77581 (19)	0.66002 (18)	0.4122 (3)	0.0573 (8)	
C3	0.8277 (2)	0.6930 (2)	0.3425 (3)	0.0691 (11)	
H3A	0.8489	0.7447	0.3558	0.083*	
C4	0.8488 (2)	0.6516 (3)	0.2543 (3)	0.0747 (11)	
H4A	0.8815	0.6757	0.2057	0.090*	
C5	0.8222 (2)	0.5757 (3)	0.2378 (3)	0.0691 (10)	
H5A	0.8391	0.5472	0.1793	0.083*	
C6	0.77098 (19)	0.5396 (2)	0.3047 (3)	0.0557 (8)	
C7	0.7496 (2)	0.4546 (2)	0.2948 (3)	0.0567 (8)	
H7A	0.7658	0.4337	0.2265	0.068*	
H7B	0.6824	0.4466	0.2868	0.068*	
C8	0.80383 (19)	0.41190 (17)	0.3989 (3)	0.0493 (7)	
C9	0.8995 (2)	0.42163 (18)	0.4304 (3)	0.0531 (7)	
H9A	0.9292	0.4513	0.3837	0.064*	
C10	0.95165 (19)	0.38909 (18)	0.5278 (3)	0.0564 (8)	
H10A	1.0170	0.3956	0.5481	0.068*	
C11	0.90806 (19)	0.34675 (18)	0.5961 (3)	0.0555 (8)	
H11A	0.9442	0.3245	0.6636	0.067*	
C12	0.8129 (2)	0.33586 (16)	0.5687 (3)	0.0516 (7)	
C13	0.76157 (18)	0.36740 (16)	0.4673 (3)	0.0459 (6)	
C14	0.7682 (2)	0.29599 (19)	0.6526 (3)	0.0672 (10)	
H14A	0.7002	0.2949	0.6225	0.081*	
H14B	0.7905	0.2420	0.6636	0.081*	
C15	0.6360 (3)	0.2828 (3)	0.3918 (5)	0.0497 (11)	0.582 (3)
H15A	0.6652	0.2422	0.4459	0.060*	0.582 (3)
H15B	0.6549	0.2746	0.3201	0.060*	0.582 (3)
C16	0.5311 (4)	0.2779 (3)	0.3707 (4)	0.0532 (10)	0.582 (3)
H16A	0.5128	0.2229	0.3587	0.064*	0.582 (3)
H16B	0.5133	0.2954	0.4398	0.064*	0.582 (3)
C17	0.4767 (4)	0.3238 (3)	0.2723 (5)	0.0580 (11)	0.582 (3)
H17A	0.4944	0.3789	0.2850	0.070*	0.582 (3)
H17D	0.4955	0.3069	0.2035	0.070*	0.582 (3)
C18	0.3738 (4)	0.3183 (4)	0.2498 (6)	0.0591 (14)	0.582 (3)
H18A	0.3462	0.3325	0.1702	0.071*	0.582 (3)
H18B	0.3562	0.2642	0.2609	0.071*	0.582 (3)
C15B	0.6356 (4)	0.3113 (4)	0.3308 (7)	0.0497 (11)	0.418 (3)
H15C	0.6513	0.2562	0.3466	0.060*	0.418 (3)
H15D	0.6675	0.3297	0.2731	0.060*	0.418 (3)
C16B	0.5309 (5)	0.3207 (4)	0.2876 (6)	0.0532 (10)	0.418 (3)
H16C	0.5172	0.3767	0.2784	0.064*	0.418 (3)
H16D	0.5124	0.2970	0.2116	0.064*	0.418 (3)
C17B	0.4721 (5)	0.2880 (5)	0.3568 (7)	0.0580 (11)	0.418 (3)
H17B	0.4868	0.3150	0.4306	0.070*	0.418 (3)
H17C	0.4895	0.2331	0.3715	0.070*	0.418 (3)
C18B	0.3699 (5)	0.2917 (5)	0.3101 (8)	0.0591 (14)	0.418 (3)
H18C	0.3548	0.2785	0.2287	0.071*	0.418 (3)
H18D	0.3389	0.2538	0.3494	0.071*	0.418 (3)
C19	0.23955 (18)	0.37101 (16)	0.3028 (3)	0.0508 (7)	
C20	0.1945 (2)	0.33526 (16)	0.3766 (3)	0.0513 (7)	
C21	0.0987 (2)	0.33485 (18)	0.3495 (3)	0.0564 (8)	
H21A	0.0670	0.3099	0.3985	0.068*	
C22	0.0481 (2)	0.3706 (2)	0.2514 (3)	0.0613 (9)	
H22A	−0.0177	0.3685	0.2324	0.074*	
C23	0.0938 (2)	0.4086 (2)	0.1829 (3)	0.0617 (9)	
H23A	0.0589	0.4341	0.1174	0.074*	
C24	0.1900 (2)	0.4110 (2)	0.2062 (3)	0.0555 (8)	
C25	0.2460 (2)	0.29988 (17)	0.4875 (3)	0.0621 (9)	
H25A	0.2293	0.2446	0.4886	0.075*	
H25B	0.3135	0.3031	0.4933	0.075*	
C26	0.2364 (2)	0.4584 (2)	0.1310 (3)	0.0735 (11)	
H26A	0.3044	0.4535	0.1581	0.088*	
H26B	0.2185	0.4378	0.0529	0.088*	
C27	0.2098 (2)	0.5424 (2)	0.1301 (3)	0.0646 (9)	
C28	0.1481 (2)	0.5766 (3)	0.0381 (3)	0.0770 (12)	
H28A	0.1263	0.5476	−0.0293	0.092*	
C29	0.1186 (2)	0.6510 (3)	0.0433 (3)	0.0811 (13)	
H29A	0.0780	0.6736	−0.0209	0.097*	
C30	0.1469 (2)	0.6929 (2)	0.1395 (3)	0.0738 (12)	
H30A	0.1245	0.7441	0.1422	0.089*	
C31	0.2087 (2)	0.6620 (2)	0.2352 (3)	0.0618 (9)	
C32	0.24148 (19)	0.5878 (2)	0.2264 (3)	0.0616 (9)	
C33	0.3978 (4)	0.5395 (3)	0.2990 (5)	0.0500 (9)	0.533 (4)
H33A	0.4286	0.4970	0.3485	0.060*	0.533 (4)
H33B	0.3931	0.5248	0.2193	0.060*	0.533 (4)
C34	0.4508 (4)	0.6121 (3)	0.3265 (5)	0.0540 (10)	0.533 (4)
H34A	0.4408	0.6322	0.3986	0.065*	0.533 (4)
H34D	0.4251	0.6506	0.2670	0.065*	0.533 (4)
C35	0.5543 (6)	0.6059 (6)	0.3371 (13)	0.055 (2)	0.533 (4)
H35A	0.5834	0.6561	0.3638	0.066*	0.533 (4)
H35B	0.5650	0.5960	0.2613	0.066*	0.533 (4)
C36	0.6019 (19)	0.5425 (19)	0.418 (2)	0.061 (3)	0.533 (4)
H36A	0.5731	0.5410	0.4830	0.073*	0.533 (4)
H36B	0.5890	0.4923	0.3782	0.073*	0.533 (4)
C33B	0.3947 (4)	0.5932 (4)	0.3494 (5)	0.0500 (9)	0.467 (4)
H33C	0.3877	0.6499	0.3401	0.060*	0.467 (4)
H33D	0.4254	0.5821	0.4293	0.060*	0.467 (4)
C34B	0.4506 (4)	0.5629 (4)	0.2750 (6)	0.0540 (10)	0.467 (4)
H34B	0.4271	0.5849	0.1984	0.065*	0.467 (4)
H34C	0.4423	0.5061	0.2690	0.065*	0.467 (4)
C35B	0.5533 (7)	0.5807 (8)	0.3147 (15)	0.055 (2)	0.467 (4)
H35C	0.5616	0.6375	0.3171	0.066*	0.467 (4)
H35D	0.5852	0.5600	0.2584	0.066*	0.467 (4)
C36B	0.600 (2)	0.547 (2)	0.431 (3)	0.061 (3)	0.467 (4)
H36C	0.5775	0.5766	0.4890	0.073*	0.467 (4)
H36D	0.5796	0.4931	0.4338	0.073*	0.467 (4)
C2B	0.5952 (3)	0.5016 (3)	0.0310 (3)	0.0789 (11)	
H2BA	0.6609	0.5034	0.0528	0.095*	
C3B	0.5466 (3)	0.5696 (3)	0.0090 (3)	0.0832 (12)	
H3BA	0.5787	0.6174	0.0148	0.100*	
C1B	0.5521 (3)	0.4318 (3)	0.0225 (3)	0.0842 (12)	
H1BA	0.5868	0.3852	0.0371	0.101*	

### Atomic displacement parameters (Å^2^)

**Table:**

	*U*^11^	*U*^22^	*U*^33^	*U*^12^	*U*^13^	*U*^23^
O1	0.0406 (10)	0.0834 (16)	0.0504 (12)	0.0035 (10)	−0.0015 (9)	0.0210 (11)
O2	0.0453 (10)	0.0508 (11)	0.0466 (11)	0.0008 (8)	0.0132 (8)	0.0061 (9)
O3	0.0388 (9)	0.0511 (11)	0.0523 (12)	−0.0009 (8)	0.0003 (8)	−0.0101 (9)
O4	0.0406 (10)	0.0584 (12)	0.0637 (14)	0.0027 (9)	−0.0025 (9)	−0.0163 (10)
C1	0.0362 (13)	0.0587 (17)	0.0468 (16)	0.0026 (11)	0.0023 (11)	0.0181 (13)
C2	0.0413 (14)	0.0564 (18)	0.066 (2)	0.0024 (12)	−0.0041 (13)	0.0259 (15)
C3	0.0461 (16)	0.069 (2)	0.082 (3)	−0.0011 (15)	−0.0058 (16)	0.042 (2)
C4	0.0524 (18)	0.108 (3)	0.060 (2)	0.0006 (19)	0.0060 (16)	0.046 (2)
C5	0.0526 (17)	0.110 (3)	0.0413 (17)	0.0066 (18)	0.0043 (14)	0.0273 (18)
C6	0.0400 (14)	0.085 (2)	0.0373 (15)	0.0043 (14)	0.0010 (11)	0.0198 (15)
C7	0.0507 (16)	0.079 (2)	0.0385 (15)	−0.0010 (15)	0.0070 (12)	−0.0100 (15)
C8	0.0463 (14)	0.0545 (16)	0.0438 (16)	0.0046 (12)	0.0046 (12)	−0.0125 (13)
C9	0.0446 (14)	0.0636 (18)	0.0504 (17)	0.0008 (13)	0.0102 (13)	−0.0051 (14)
C10	0.0358 (14)	0.0645 (19)	0.064 (2)	0.0009 (13)	0.0032 (13)	−0.0028 (16)
C11	0.0440 (14)	0.0493 (16)	0.065 (2)	0.0010 (13)	−0.0035 (14)	0.0053 (14)
C12	0.0454 (14)	0.0413 (14)	0.0619 (19)	−0.0020 (11)	0.0009 (13)	0.0011 (13)
C13	0.0386 (13)	0.0436 (14)	0.0505 (16)	−0.0002 (11)	0.0008 (11)	−0.0098 (12)
C14	0.0467 (16)	0.0586 (19)	0.085 (3)	−0.0114 (14)	−0.0074 (16)	0.0227 (18)
C15	0.0473 (19)	0.036 (2)	0.058 (3)	−0.0012 (18)	−0.002 (2)	−0.0046 (18)
C16	0.054 (2)	0.050 (2)	0.048 (2)	−0.006 (2)	−0.001 (2)	−0.0067 (17)
C17	0.049 (2)	0.061 (3)	0.059 (3)	0.004 (2)	0.003 (2)	−0.003 (2)
C18	0.048 (2)	0.059 (4)	0.067 (4)	0.011 (2)	0.009 (3)	−0.014 (3)
C15B	0.0473 (19)	0.036 (2)	0.058 (3)	−0.0012 (18)	−0.002 (2)	−0.0046 (18)
C16B	0.054 (2)	0.050 (2)	0.048 (2)	−0.006 (2)	−0.001 (2)	−0.0067 (17)
C17B	0.049 (2)	0.061 (3)	0.059 (3)	0.004 (2)	0.003 (2)	−0.003 (2)
C18B	0.048 (2)	0.059 (4)	0.067 (4)	0.011 (2)	0.009 (3)	−0.014 (3)
C19	0.0403 (14)	0.0467 (15)	0.0576 (18)	0.0043 (11)	−0.0032 (13)	−0.0185 (13)
C20	0.0488 (15)	0.0410 (15)	0.0584 (18)	0.0012 (12)	0.0018 (13)	−0.0105 (13)
C21	0.0490 (15)	0.0551 (18)	0.0598 (19)	0.0039 (13)	0.0030 (14)	−0.0102 (15)
C22	0.0389 (14)	0.085 (2)	0.0549 (19)	0.0049 (14)	0.0009 (13)	−0.0135 (17)
C23	0.0460 (16)	0.086 (2)	0.0461 (17)	0.0164 (15)	−0.0026 (14)	−0.0141 (16)
C24	0.0485 (16)	0.070 (2)	0.0447 (17)	0.0037 (14)	0.0043 (13)	−0.0140 (14)
C25	0.0463 (15)	0.0436 (16)	0.088 (3)	0.0006 (12)	−0.0001 (16)	0.0109 (16)
C26	0.0520 (18)	0.126 (3)	0.0403 (17)	0.0173 (19)	0.0073 (14)	−0.0030 (19)
C27	0.0454 (16)	0.104 (3)	0.0426 (17)	0.0038 (16)	0.0069 (13)	0.0215 (18)
C28	0.0524 (18)	0.130 (4)	0.0441 (18)	0.002 (2)	0.0025 (15)	0.026 (2)
C29	0.0578 (19)	0.111 (3)	0.062 (2)	−0.010 (2)	−0.0089 (17)	0.047 (2)
C30	0.0516 (17)	0.083 (2)	0.078 (3)	−0.0124 (16)	−0.0023 (17)	0.048 (2)
C31	0.0421 (14)	0.071 (2)	0.064 (2)	−0.0143 (14)	−0.0024 (13)	0.0342 (17)
C32	0.0358 (14)	0.092 (3)	0.0531 (19)	−0.0039 (14)	0.0035 (13)	0.0313 (18)
C33	0.049 (2)	0.053 (2)	0.045 (2)	0.003 (2)	0.0049 (19)	−0.0055 (17)
C34	0.052 (2)	0.061 (3)	0.050 (3)	−0.003 (2)	0.013 (2)	0.0011 (19)
C35	0.0464 (17)	0.060 (7)	0.055 (6)	0.000 (3)	0.007 (2)	0.010 (5)
C36	0.0468 (18)	0.076 (4)	0.060 (5)	−0.0029 (19)	0.013 (3)	0.014 (4)
C33B	0.049 (2)	0.053 (2)	0.045 (2)	0.003 (2)	0.0049 (19)	−0.0055 (17)
C34B	0.052 (2)	0.061 (3)	0.050 (3)	−0.003 (2)	0.013 (2)	0.0011 (19)
C35B	0.0464 (17)	0.060 (7)	0.055 (6)	0.000 (3)	0.007 (2)	0.010 (5)
C36B	0.0468 (18)	0.076 (4)	0.060 (5)	−0.0029 (19)	0.013 (3)	0.014 (4)
C2B	0.064 (2)	0.123 (3)	0.0475 (19)	−0.002 (2)	0.0073 (17)	0.005 (2)
C3B	0.082 (3)	0.124 (4)	0.0413 (18)	−0.001 (2)	0.0098 (17)	−0.001 (2)
C1B	0.083 (3)	0.117 (4)	0.048 (2)	−0.001 (2)	0.0080 (19)	−0.004 (2)

### Geometric parameters (Å, º)

**Table:**

O1—C32	1.379 (3)	C18B—H18C	0.9900
O1—C33B	1.492 (7)	C18B—H18D	0.9900
O1—C33	1.525 (6)	C19—C20	1.390 (5)
O2—C1	1.375 (3)	C19—C24	1.411 (4)
O2—C36	1.41 (3)	C20—C21	1.383 (4)
O2—C36B	1.42 (3)	C20—C25	1.513 (4)
O3—C13	1.393 (3)	C21—C22	1.395 (5)
O3—C15	1.440 (5)	C21—H21A	0.9500
O3—C15B	1.500 (7)	C22—C23	1.366 (5)
O4—C19	1.401 (3)	C22—H22A	0.9500
O4—C18B	1.461 (8)	C23—C24	1.391 (4)
O4—C18	1.478 (6)	C23—H23A	0.9500
C1—C6	1.398 (5)	C24—C26	1.516 (5)
C1—C2	1.402 (4)	C25—C2^i^	1.508 (5)
C2—C3	1.400 (5)	C25—H25A	0.9900
C2—C25^i^	1.508 (5)	C25—H25B	0.9900
C3—C4	1.389 (6)	C26—C27	1.505 (5)
C3—H3A	0.9500	C26—H26A	0.9900
C4—C5	1.372 (6)	C26—H26B	0.9900
C4—H4A	0.9500	C27—C32	1.397 (5)
C5—C6	1.391 (5)	C27—C28	1.399 (4)
C5—H5A	0.9500	C28—C29	1.366 (6)
C6—C7	1.502 (5)	C28—H28A	0.9500
C7—C8	1.522 (4)	C29—C30	1.356 (6)
C7—H7A	0.9900	C29—H29A	0.9500
C7—H7B	0.9900	C30—C31	1.406 (4)
C8—C13	1.392 (4)	C30—H30A	0.9500
C8—C9	1.392 (4)	C31—C32	1.386 (5)
C9—C10	1.374 (5)	C31—C14^i^	1.513 (5)
C9—H9A	0.9500	C33—C34	1.478 (7)
C10—C11	1.382 (5)	C33—H33A	0.9900
C10—H10A	0.9500	C33—H33B	0.9900
C11—C12	1.387 (4)	C34—C35	1.518 (10)
C11—H11A	0.9500	C34—H34A	0.9900
C12—C13	1.398 (4)	C34—H34D	0.9900
C12—C14	1.514 (5)	C35—C36	1.528 (13)
C14—C31^i^	1.513 (5)	C35—H35A	0.9900
C14—H14A	0.9900	C35—H35B	0.9900
C14—H14B	0.9900	C36—H36A	0.9900
C15—C16	1.521 (7)	C36—H36B	0.9900
C15—H15A	0.9900	C33B—C34B	1.467 (8)
C15—H15B	0.9900	C33B—H33C	0.9900
C16—C17	1.503 (7)	C33B—H33D	0.9900
C16—H16A	0.9900	C34B—C35B	1.519 (11)
C16—H16B	0.9900	C34B—H34B	0.9900
C17—C18	1.492 (7)	C34B—H34C	0.9900
C17—H17A	0.9900	C35B—C36B	1.531 (15)
C17—H17D	0.9900	C35B—H35C	0.9900
C18—H18A	0.9900	C35B—H35D	0.9900
C18—H18B	0.9900	C36B—H36C	0.9900
C15B—C16B	1.529 (9)	C36B—H36D	0.9900
C15B—H15C	0.9900	C2B—C1B	1.361 (6)
C15B—H15D	0.9900	C2B—C3B	1.372 (6)
C16B—C17B	1.466 (10)	C2B—H2BA	0.9500
C16B—H16C	0.9900	C3B—C1B^ii^	1.426 (6)
C16B—H16D	0.9900	C3B—H3BA	0.9500
C17B—C18B	1.492 (9)	C1B—C3B^ii^	1.426 (6)
C17B—H17B	0.9900	C1B—H1BA	0.9500
C17B—H17C	0.9900		
			
C32—O1—C33B	114.1 (3)	C20—C19—C24	121.4 (3)
C32—O1—C33	113.5 (3)	O4—C19—C24	118.6 (3)
C1—O2—C36	117.2 (12)	C21—C20—C19	118.5 (3)
C1—O2—C36B	121.7 (12)	C21—C20—C25	118.9 (3)
C13—O3—C15	114.1 (3)	C19—C20—C25	122.6 (3)
C13—O3—C15B	111.6 (3)	C20—C21—C22	121.0 (3)
C19—O4—C18B	111.0 (4)	C20—C21—H21A	119.5
C19—O4—C18	114.5 (3)	C22—C21—H21A	119.5
O2—C1—C6	119.6 (3)	C23—C22—C21	119.4 (3)
O2—C1—C2	117.9 (3)	C23—C22—H22A	120.3
C6—C1—C2	122.4 (3)	C21—C22—H22A	120.3
C3—C2—C1	117.1 (4)	C22—C23—C24	121.9 (3)
C3—C2—C25^i^	124.2 (3)	C22—C23—H23A	119.0
C1—C2—C25^i^	118.5 (3)	C24—C23—H23A	119.0
C4—C3—C2	121.3 (4)	C23—C24—C19	117.5 (3)
C4—C3—H3A	119.3	C23—C24—C26	119.3 (3)
C2—C3—H3A	119.3	C19—C24—C26	123.2 (3)
C5—C4—C3	119.7 (3)	C2^i^—C25—C20	112.1 (2)
C5—C4—H4A	120.1	C2^i^—C25—H25A	109.2
C3—C4—H4A	120.1	C20—C25—H25A	109.2
C4—C5—C6	121.6 (4)	C2^i^—C25—H25B	109.2
C4—C5—H5A	119.2	C20—C25—H25B	109.2
C6—C5—H5A	119.2	H25A—C25—H25B	107.9
C5—C6—C1	117.7 (3)	C27—C26—C24	111.6 (3)
C5—C6—C7	121.9 (3)	C27—C26—H26A	109.3
C1—C6—C7	120.1 (3)	C24—C26—H26A	109.3
C6—C7—C8	110.4 (2)	C27—C26—H26B	109.3
C6—C7—H7A	109.6	C24—C26—H26B	109.3
C8—C7—H7A	109.6	H26A—C26—H26B	108.0
C6—C7—H7B	109.6	C32—C27—C28	117.3 (4)
C8—C7—H7B	109.6	C32—C27—C26	120.2 (3)
H7A—C7—H7B	108.1	C28—C27—C26	122.4 (4)
C13—C8—C9	118.8 (3)	C29—C28—C27	121.2 (4)
C13—C8—C7	122.9 (3)	C29—C28—H28A	119.4
C9—C8—C7	118.2 (3)	C27—C28—H28A	119.4
C10—C9—C8	121.1 (3)	C30—C29—C28	120.5 (3)
C10—C9—H9A	119.5	C30—C29—H29A	119.8
C8—C9—H9A	119.5	C28—C29—H29A	119.8
C9—C10—C11	119.3 (3)	C29—C30—C31	121.3 (4)
C9—C10—H10A	120.4	C29—C30—H30A	119.3
C11—C10—H10A	120.4	C31—C30—H30A	119.3
C10—C11—C12	121.8 (3)	C32—C31—C30	117.3 (4)
C10—C11—H11A	119.1	C32—C31—C14^i^	120.5 (3)
C12—C11—H11A	119.1	C30—C31—C14^i^	122.0 (4)
C11—C12—C13	118.0 (3)	O1—C32—C31	119.3 (3)
C11—C12—C14	119.2 (3)	O1—C32—C27	118.3 (3)
C13—C12—C14	122.6 (3)	C31—C32—C27	122.2 (3)
C8—C13—O3	119.1 (2)	C34—C33—O1	106.3 (4)
C8—C13—C12	121.1 (2)	C34—C33—H33A	110.5
O3—C13—C12	119.8 (3)	O1—C33—H33A	110.5
C31^i^—C14—C12	110.5 (2)	C34—C33—H33B	110.5
C31^i^—C14—H14A	109.6	O1—C33—H33B	110.5
C12—C14—H14A	109.6	H33A—C33—H33B	108.7
C31^i^—C14—H14B	109.6	C33—C34—C35	115.7 (6)
C12—C14—H14B	109.6	C33—C34—H34A	108.3
H14A—C14—H14B	108.1	C35—C34—H34A	108.3
O3—C15—C16	109.0 (4)	C33—C34—H34D	108.3
O3—C15—H15A	109.9	C35—C34—H34D	108.3
C16—C15—H15A	109.9	H34A—C34—H34D	107.4
O3—C15—H15B	109.9	C34—C35—C36	114.2 (13)
C16—C15—H15B	109.9	C34—C35—H35A	108.7
H15A—C15—H15B	108.3	C36—C35—H35A	108.7
C17—C16—C15	116.0 (5)	C34—C35—H35B	108.7
C17—C16—H16A	108.3	C36—C35—H35B	108.7
C15—C16—H16A	108.3	H35A—C35—H35B	107.6
C17—C16—H16B	108.3	O2—C36—C35	117 (2)
C15—C16—H16B	108.3	O2—C36—H36A	108.1
H16A—C16—H16B	107.4	C35—C36—H36A	108.1
C18—C17—C16	116.4 (5)	O2—C36—H36B	108.1
C18—C17—H17A	108.2	C35—C36—H36B	108.1
C16—C17—H17A	108.2	H36A—C36—H36B	107.3
C18—C17—H17D	108.2	C34B—C33B—O1	108.2 (5)
C16—C17—H17D	108.2	C34B—C33B—H33C	110.1
H17A—C17—H17D	107.3	O1—C33B—H33C	110.1
O4—C18—C17	111.8 (5)	C34B—C33B—H33D	110.1
O4—C18—H18A	109.3	O1—C33B—H33D	110.1
C17—C18—H18A	109.3	H33C—C33B—H33D	108.4
O4—C18—H18B	109.3	C33B—C34B—C35B	114.0 (7)
C17—C18—H18B	109.3	C33B—C34B—H34B	108.7
H18A—C18—H18B	107.9	C35B—C34B—H34B	108.7
O3—C15B—C16B	108.0 (5)	C33B—C34B—H34C	108.7
O3—C15B—H15C	110.1	C35B—C34B—H34C	108.7
C16B—C15B—H15C	110.1	H34B—C34B—H34C	107.6
O3—C15B—H15D	110.1	C34B—C35B—C36B	114.5 (15)
C16B—C15B—H15D	110.1	C34B—C35B—H35C	108.6
H15C—C15B—H15D	108.4	C36B—C35B—H35C	108.6
C17B—C16B—C15B	117.4 (7)	C34B—C35B—H35D	108.6
C17B—C16B—H16C	108.0	C36B—C35B—H35D	108.6
C15B—C16B—H16C	108.0	H35C—C35B—H35D	107.6
C17B—C16B—H16D	108.0	O2—C36B—C35B	115 (2)
C15B—C16B—H16D	108.0	O2—C36B—H36C	108.4
H16C—C16B—H16D	107.2	C35B—C36B—H36C	108.4
C16B—C17B—C18B	117.3 (7)	O2—C36B—H36D	108.4
C16B—C17B—H17B	108.0	C35B—C36B—H36D	108.4
C18B—C17B—H17B	108.0	H36C—C36B—H36D	107.5
C16B—C17B—H17C	108.0	C1B—C2B—C3B	122.0 (4)
C18B—C17B—H17C	108.0	C1B—C2B—H2BA	119.0
H17B—C17B—H17C	107.2	C3B—C2B—H2BA	119.0
O4—C18B—C17B	109.8 (6)	C2B—C3B—C1B^ii^	119.9 (5)
O4—C18B—H18C	109.7	C2B—C3B—H3BA	120.0
C17B—C18B—H18C	109.7	C1B^ii^—C3B—H3BA	120.0
O4—C18B—H18D	109.7	C2B—C1B—C3B^ii^	118.1 (5)
C17B—C18B—H18D	109.7	C2B—C1B—H1BA	121.0
H18C—C18B—H18D	108.2	C3B^ii^—C1B—H1BA	121.0
C20—C19—O4	119.9 (3)		
			
C36—O2—C1—C6	−81.0 (18)	C18—O4—C19—C20	107.8 (4)
C36B—O2—C1—C6	−88 (2)	C18B—O4—C19—C24	−111.5 (5)
C36—O2—C1—C2	102.7 (17)	C18—O4—C19—C24	−73.8 (4)
C36B—O2—C1—C2	95 (2)	O4—C19—C20—C21	−177.2 (2)
O2—C1—C2—C3	178.3 (2)	C24—C19—C20—C21	4.5 (4)
C6—C1—C2—C3	2.0 (4)	O4—C19—C20—C25	5.5 (4)
O2—C1—C2—C25^i^	1.7 (4)	C24—C19—C20—C25	−172.8 (3)
C6—C1—C2—C25^i^	−174.6 (2)	C19—C20—C21—C22	−1.1 (5)
C1—C2—C3—C4	0.7 (4)	C25—C20—C21—C22	176.3 (3)
C25^i^—C2—C3—C4	177.1 (3)	C20—C21—C22—C23	−2.0 (5)
C2—C3—C4—C5	−3.0 (5)	C21—C22—C23—C24	1.9 (5)
C3—C4—C5—C6	2.6 (5)	C22—C23—C24—C19	1.4 (5)
C4—C5—C6—C1	0.1 (4)	C22—C23—C24—C26	−176.2 (3)
C4—C5—C6—C7	−173.9 (3)	C20—C19—C24—C23	−4.6 (4)
O2—C1—C6—C5	−178.6 (2)	O4—C19—C24—C23	177.0 (3)
C2—C1—C6—C5	−2.5 (4)	C20—C19—C24—C26	172.8 (3)
O2—C1—C6—C7	−4.6 (4)	O4—C19—C24—C26	−5.5 (4)
C2—C1—C6—C7	171.6 (3)	C21—C20—C25—C2^i^	−60.7 (4)
C5—C6—C7—C8	109.6 (3)	C19—C20—C25—C2^i^	116.6 (3)
C1—C6—C7—C8	−64.2 (3)	C23—C24—C26—C27	59.7 (4)
C6—C7—C8—C13	122.4 (3)	C19—C24—C26—C27	−117.7 (3)
C6—C7—C8—C9	−53.7 (4)	C24—C26—C27—C32	69.7 (4)
C13—C8—C9—C10	−0.9 (5)	C24—C26—C27—C28	−105.5 (4)
C7—C8—C9—C10	175.3 (3)	C32—C27—C28—C29	−1.4 (5)
C8—C9—C10—C11	−0.7 (5)	C26—C27—C28—C29	174.0 (3)
C9—C10—C11—C12	0.3 (5)	C27—C28—C29—C30	−1.7 (6)
C10—C11—C12—C13	1.7 (5)	C28—C29—C30—C31	1.5 (6)
C10—C11—C12—C14	−173.8 (3)	C29—C30—C31—C32	1.8 (5)
C9—C8—C13—O3	179.4 (3)	C29—C30—C31—C14^i^	−172.4 (3)
C7—C8—C13—O3	3.3 (4)	C33B—O1—C32—C31	−67.4 (4)
C9—C8—C13—C12	3.0 (4)	C33—O1—C32—C31	−115.3 (4)
C7—C8—C13—C12	−173.1 (3)	C33B—O1—C32—C27	117.5 (4)
C15—O3—C13—C8	105.6 (4)	C33—O1—C32—C27	69.6 (4)
C15B—O3—C13—C8	67.4 (4)	C30—C31—C32—O1	−179.8 (3)
C15—O3—C13—C12	−78.0 (4)	C14^i^—C31—C32—O1	−5.6 (4)
C15B—O3—C13—C12	−116.2 (4)	C30—C31—C32—C27	−5.0 (5)
C11—C12—C13—C8	−3.4 (4)	C14^i^—C31—C32—C27	169.3 (3)
C14—C12—C13—C8	171.9 (3)	C28—C27—C32—O1	179.7 (3)
C11—C12—C13—O3	−179.7 (3)	C26—C27—C32—O1	4.2 (5)
C14—C12—C13—O3	−4.4 (4)	C28—C27—C32—C31	4.8 (5)
C11—C12—C14—C31^i^	56.5 (4)	C26—C27—C32—C31	−170.7 (3)
C13—C12—C14—C31^i^	−118.8 (3)	C32—O1—C33—C34	85.6 (5)
C13—O3—C15—C16	175.6 (4)	C33B—O1—C33—C34	−15.3 (5)
C15B—O3—C15—C16	−90.7 (7)	O1—C33—C34—C35	167.7 (8)
O3—C15—C16—C17	72.4 (6)	C33—C34—C35—C36	−52.3 (18)
C15—C16—C17—C18	179.0 (5)	C1—O2—C36—C35	−32 (3)
C19—O4—C18—C17	175.6 (4)	C36B—O2—C36—C35	95 (19)
C18B—O4—C18—C17	−92.3 (8)	C34—C35—C36—O2	−160 (2)
C16—C17—C18—O4	80.8 (6)	C32—O1—C33B—C34B	−81.5 (6)
C13—O3—C15B—C16B	−166.0 (5)	C33—O1—C33B—C34B	17.9 (5)
C15—O3—C15B—C16B	92.3 (8)	O1—C33B—C34B—C35B	−165.5 (8)
O3—C15B—C16B—C17B	−66.8 (8)	C33B—C34B—C35B—C36B	60 (2)
C15B—C16B—C17B—C18B	−175.3 (7)	C1—O2—C36B—C35B	4 (4)
C19—O4—C18B—C17B	−168.4 (6)	C36—O2—C36B—C35B	−53 (16)
C18—O4—C18B—C17B	88.7 (9)	C34B—C35B—C36B—O2	168 (2)
C16B—C17B—C18B—O4	−78.3 (9)	C1B—C2B—C3B—C1B^ii^	0.8 (7)
C18B—O4—C19—C20	70.1 (5)	C3B—C2B—C1B—C3B^ii^	−0.7 (7)

Symmetry codes: (i) −*x*+1, −*y*+1, −*z*+1; (ii) −*x*+1, −*y*+1, −*z*.
